# Adaptive and innate immune mechanisms in cardiac fibrosis complicating pulmonary arterial hypertension

**DOI:** 10.14814/phy2.14532

**Published:** 2020-08-12

**Authors:** Jamila H. Siamwala, Alexander Zhao, Haley Barthel, Francesco S. Pagano, Richard J. Gilbert, Sharon Rounds

**Affiliations:** ^1^ Department of Molecular Pharmacology Physiology and Biotechnology Brown University Providence RI USA; ^2^ Warren Alpert Medical School of Brown University Providence VA Medical Center Providence RI USA; ^3^ Ocean State Research Institute Providence VA Medical Center Providence RI USA; ^4^ Department of Medicine Division of Pulmonary Critical Care and Sleep Warren Alpert Medical School of Brown University Providence RI USA

**Keywords:** heart failure, hypertrophy, inflammation, innate immunity, interleukin‐1 beta, pulmonary arterial hypertension, right ventricular fibrosis, tissue resident macrophages

## Abstract

Pulmonary arterial hypertension (PAH) is a syndrome diagnosed by increased mean pulmonary artery (PA) pressure and resistance and normal pulmonary capillary wedge pressure. PAH is characterized pathologically by distal pulmonary artery remodeling, increased pulmonary vascular resistance, and plexiform lesions (PLs). Right ventricular fibrosis and hypertrophy, leading to right ventricular failure, are the main determinants of mortality in PAH. Recent work suggests that right ventricular fibrosis results from resident cardiac fibroblast activation and conversion to myofibroblasts, leading to replacement of contractile cardiomyocytes with nondistensible tissue incapable of conductivity or contractility. However, the origins, triggers, and consequences of myofibroblast expansion and its pathophysiological relationship with PAH are unclear. Recent advances indicate that signals generated by adaptive and innate immune cells may play a role in right ventricular fibrosis and remodeling. This review summarizes recent insights into the mechanisms by which adaptive and innate immune signals participate in the transition of cardiac fibroblasts to activated myofibroblasts and highlights the existing gaps of knowledge as relates to the development of right ventricular fibrosis.

## Graphical abstract

Adaptive and maladaptive cardiac remodeling results from the secretion of pro‐inflammatory and profibrotic cytokines and chemokines originating in macrophages (innate immune responses) and helper T cells (adaptive immunity). Direct conversion of one cell type to another may also be a mechanism of disease pathology in the setting of PAH.

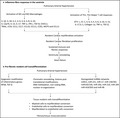

## 1 INTRODUCTION

Pulmonary arterial hypertension (PAH) or Group I Pulmonary Hypertension is a severe debilitating syndrome of multiple etiologies including connective tissue disorders, drugs, toxins, and heritable causes with a 5‐year overall survival rate of 65% of the affected individuals in spite of advanced therapies (Farber et al., 2015).The prevalence of PAH worldwide is estimated to be between 6.6 and 26 cases per million individuals (Hoeper et al., 2016) and the incidence of PAH according to the Registry to Evaluate Early and Long‐Term Pulmonary Arterial Hypertension Disease Management (REVEAL) registry is 2.4 per million of the adult population in the United States (Frost et al., 2011). PAH affects people of all geographic locations, ages, races, and sex (Brown et al., 2011), although females are ~ 2 to 3 times more likely to develop PAH compared to males (Frost et al., (2011)). Male patients diagnosed with PAH are older than female patients at the time of diagnosis and have poorer outcomes (Maron & Galiè, 2016). PAH is clinically defined as a syndrome of resting mean pulmonary arterial pressure ≥25 mmHg measured by right heart catheterization (Hatano & Strasser, 1975; Hoeper et al., 2013), although a recent World Symposium on Pulmonary Hypertension suggested redefining adult Group 1 PAH as a mean resting pulmonary arterial pressure ≥20 mmHg with pulmonary vascular resistance ≥3 Wood Units and pulmonary artery wedge pressure ≤15 mmHg (Simonneau et al., 2018). Symptoms of PAH include dyspnea, fatigue, weakness, angina, syncope, peripheral edema, and abdominal distension, (Barst et al., 2004) and there is currently no curative treatment.

Since right ventricular failure is the usual cause of mortality in PAH, this review summarizes knowledge regarding adaptive and innate immune mechanisms and the development of right ventricular fibrosis in PAH and relates this to growing knowledge of the role of immune mechanisms and fibrosis in left ventricular failure.

## 2 PATHOPHYSIOLOGY

PAH is characterized pathologically by excessive vascular remodeling of the intima, media, and adventitial layers of the distal pulmonary precapillary arterioles arising from accelerated proliferation of smooth muscle cells, pulmonary endothelial cells and fibroblasts (Rabinovitch, 2012), leading to obstruction of the pulmonary vessels and elevated pulmonary vascular resistance (Humbert, Sitbon, & Simonneau, 2004). Additionally, there is rarefication of distal vessels which can increase pressure and reduce lung perfusion. This pulmonary vasculopathy leads to increased pulmonary vascular resistance, right ventricular pressure overload, right ventricular hypertrophy, right ventricular failure, and death (Vonk Noordegraaf & Galie, 2011). The thin‐walled right ventricle initially accommodates increases in pressure with increased contractility, hypertrophy, and homeometric adaptation without change in the dimensions of the heart. However, rapid increases in pulmonary arterial pressure lead to maladaptive remodeling of the right ventricle, right heart dilatation, stiffening of the ventricular wall, and subsequent heart failure (Naeije, 2015; Naeije & Manes, 2014). Until recently, the right ventricle has been a poorly understood and understudied cardiac structure.

The right ventricle originates from the secondary heart field with lower oxygen demand compared to the left ventricle (Friedberg & Redington, 2014). The crescent‐shaped thin walled right ventricle of the heart can be easily fatigued since right ventricular mechanical function is solely dependent on ventricular preload and afterload. To compensate for increases in right ventricular pressure afterload, the highly compliant right ventricle adapts rapidly to changes in volume load by enhancing contractility, thus causing right ventricular hypertrophy (Naeije & Manes, 2014). The left ventricle may also fail in PAH as the dilated right ventricle impairs filling of the left ventricular cavity, with resultant reduction in the left ventricular ejection fraction, contractility, and maximum force‐generating capability (Manders et al., 2014). Under normal conditions, the right ventricle is much smaller than the left ventricle, however, in the setting of PAH, increases in systolic and diastolic pressures cause concentric right ventricle hypertrophy. The increases in right ventricular systolic and diastolic pressures further lead to increased muscle mass through protein synthesis and cardiomyocyte hypertrophy. Adaptive hypertrophy of the right ventricle is not maintained in the presence of chronic pressure increases, ultimately leading to right ventricular decompensation, impaired diastolic function, and low cardiac output (Vonk Noordegraaf & Galie, 2011). There is no clear consensus on whether the alterations in the right ventricle are adaptive or maladaptive and on the mechanism(s) involved in the progression to heart failure (Badagliacca et al., 2016; Rich et al., 2010; Ryan et al., 2015; Veerdonk, Bogaard, & Voelkel, 2016). Animal models of PAH suggest that alterations in the right ventricular perfusion, metabolism, vessel rarefaction, and right ventricular fibrosis contribute to the switch from adaptive to maladaptive right ventricular hypertrophy and failure (Rain et al., 2016; Thenappan, Ormiston, Ryan, & Archer, 2018). pathological right ventricular fibrosis characterized by uncontrolled cardiac fibroblast proliferation and excess fibrillary collagen deposition causes increased right ventricular diastolic stiffness associated with impaired diastolic function and clinical progression of PAH in human patients (Trip et al., 2015). In patients with PAH, circulating markers of collagen metabolism indicating fibrotic responses and vascular narrowing indices are correlated with a higher mortality (Golob, Wang, Prostrollo, Hacker, & Chesler, 2016; Safdar et al., 2014; Vanderpool et al., 2015). PAH associated with connective tissue diseases, such as systemic sclerosis, has a poor prognosis with a 5‐year overall survival rate of 50% in affected individuals (Safdar et al., 2014; Trip et al., 2015). The pro‐fibrotic mediators and resident cardiac fibroblast activators involved in the switch from adaptive to maladaptive right ventricular remodeling, decompensation, decreased contractility and cardiac output, systolic and diastolic dysfunction are unknown.

## 3 IMMUNE CELL PATHOGENESIS OF CARDIAC FIBROSIS

Most literature to date has focused on the pathogenesis of left ventricular fibrosis, and considerably less is known regarding the pathogenesis of right ventricular fibrosis, including both nonimmune and immune cell mechanisms that may contribute to inflammation mediated fibrosis.

The most common connective tissue cell types in the myocardium are the resident cardiac fibroblasts or differentiated myofibroblasts that provide structural support and promote conductivity. Cardiac fibroblasts are morphologically and structurally diverse consisting of dynamic, nonmyocyte populations residing in the epicardium. These cells regulate tissue homeostasis and participate in repair and regeneration after an ischemic/reperfusion or pressure overload event. The contractile cardiac fibroblasts are heterogeneous in function and secrete extracellular matrix upon activation by a spectrum of stress signals and inflammatory signals to prevent ventricular rupture (Ivey & Tallquist, 2016).

Cardiac fibroblasts migrate, proliferate, and differentiate in response to autocrine and paracrine signals and are implicated both in tissue regeneration and pathology (Souders, Bowers, & Baudino, 2009). Cardiac fibroblasts show chamber‐specific proliferative response. The atrial cardiac fibroblasts proliferate at a rate three folds higher than ventricular cardiac fibroblasts and this mechanisms is believed to protect the atria while the ventricle undergoes pressure or volume associated remodeling (Rizvi et al., 2016). Furthermore, a recent paper indicates that cyclic overstretch and aldosterone modulates pro‐proliferative and profibrotic activators, mi‐R21 and miR‐221 in the RV but not the LV in vitro and in vivo (Powers et al., 2020).

Pro‐inflammatory cytokines from cardiac fibroblast cells, innate immune cells, and vascular cells have been identified that may contribute to fibrotic responses through varied mechanisms. Activated cardiac fibroblasts produce mediators such as monocyte recruitment factors (MCP‐1), growth factors, cytokines, and proteases that are involved in tissue repair and regeneration (desJardins‐Park HE, Foster DS, & Longaker MT., 2018; Furtado, Nim, Boyd, & Rosenthal, 2016). The sites of action of these tissue repair and regeneration mediators and their role in fibrosis are poorly understood. Lineage tracing studies are unable to fully identify cardiac fibroblasts types because the fibroblasts exhibit distinct phenotypic markers depending on the niche and microenvironmental cues. Typical fibroblast markers such as, collagen 1α transcription factor TCF21, and membrane receptor PDGFRα are expressed by other cell types contributing to the ambiguity of the identity of fibroblasts (Tallquist, 2018; Tallquist & Molkentin, 2017). The activation states and fate of the fibroblasts in right ventricular pressure overload conditions such as PAH are unknown.

Additionally, exogenous noxious stimuli, such as cigarette smoke, may promote the proliferation of rat cardiac fibroblasts, acting through α7 nicotinic acetylcholine receptors and various protein kinases (Vang et al., 2017). In the event of cardiac injury, fibroblasts differentiate into myofibroblasts expressing alpha smooth muscle actin with rapid enlargement of Golgi apparatus. The myofibroblasts initiate tissue reconstruction by producing extracellular matrix and transmitting signals to neighboring cardiomyocytes and stromal cells (Frangogiannis, 2017; Furtado et al., 2016; Pellman, Zhang, & Sheikh, 2016; Shinde & Frangogiannis, 2014). In right ventricular fibrosis, dysregulated fibroblast behavior in response to unknown extrinsic factor(s) leads to matrix deposition and scar formation (Choudhary, Troncales, Martin, Harrington, & Klinger, 2011; Schreier, Hacker, Song, & Chesler, 2013; Wynn, 2007). The mediators and mechanisms underlying the transition of cardiac fibroblasts to myofibroblasts or the fate of myofibroblasts beyond scar tissue formation in the settings of right ventricular afterload are unclear. We discuss below several specific mechanisms by which immune cells may participate in the process of ventricular fibrosis and failure, with emphasis on right‐sided ventricular disease when possible.

## 4 TRANSDIFFERENTIATION OF IMMUNE CELLS INTO MYOFIBROBLASTS

Myeloid cells or wound macrophages transdifferentiate into cardiac myofibroblast cells in response to miR‐21 (Sinha et al., 2018). The sudden surge in the number of myofibroblasts in the ventricle is in response to pressure overload is highly debated. One of the paradigms is that myeloid cells are a source of the myofibroblasts recruited to the ischemic regions and adds to the resident myofibroblast pool following myocardial injury (Sinha et al., 2018). Resident cells such as epidermal and endothelial cells transdifferentiate into myofibroblasts adding to the heterogeneity (He et al., 2017). Cardiac fibroblasts can also be reprogrammed into cardiomyocytes using specific factors suggesting the versatility and plasticity (Fu & Srivastava, 2015; Lu et al., 2020). Researchers have also shown the transdifferentiation of fibroblasts into endothelial cells to recapillarize the tissue (Sayed et al., 2015).

Irrespective of the origin of the increased myofibroblast population, it is commonly agreed that the activated, secretory interstitial, and perivascular cardiac myofibroblasts are the principal drivers of cardiac fibrosis through excess fibrillary collagen Iα deposition and right ventricular maladaptive remodeling (Tallquist & Molkentin, 2017). The numbers of cardiac fibroblasts vary among species, with mice having the lowest percentage of cardiac fibroblasts and the humans having the largest percentage of cardiac fibroblasts, which may correlate with inter‐species heterogeneity in fibrotic responses.

The transdifferentiation of immune cells, endothelial cells, and epithelial cells into cardiac myofibroblast cells in the left ventricle to secrete cytokines and extracellular matrix is disputed as transdifferentiation offers no explanation for the role of resident cardiac fibroblasts (Zeisberg & Kalluri, 2010). We hypothesize that sufficient energy, nutrients, and cell communication are required to cause transdifferentiation by rechanneling of resources from other regions of the heart and from other organs. Therefore, in‐depth study of knocking out specific noncardiac fibroblast populations and studying the cardiac myofibroblast number in homeostasis and disease conditions is required. In a genetic periostin conditional knockout mouse model, newly activated fibroblast deletion leads to insufficient collagen production and ventricular rupture emphasizing the significance of activated fibroblasts in tissue healing (Kanisicak et al., 2016). Another study showed similar results in preventing adverse cardiac remodeling in mice when removing the periostin‐expressing activated fibroblasts (Kaur et al., 2016).

High resolution, comprehensive gene expression profiling of mouse heart nonmyocytes indicates cellular diversity of “nonmyocytes” and their subpopulations. Added to the complexity are sex‐specific cardiac gene expression and the emergence of a “fibrocyte” population defined as a subpopulation of cells that exhibit hybrid features of macrophages and fibroblasts (Skelly et al., 2018). Further single cell RNAseq on total cardiac nonmyocyte populations and enriched fibroblast lineages (Platelet derived growth factor receptor [PDGFR]α^‐^GFP^+^ populations) in sham operated and 3 and 7 days post myocardial infarction models show previously unidentified lineages among the fibroblasts (Farbehi et al., 2019). Novel subtypes of myofibroblasts with characteristic pro‐ or anti‐fibrotic signatures have also been described (Farbehi et al., 2019). Cardiac fibroblast cells may have greater plasticity than previously recognized in that they may transdifferentiate into different cell populations with more than a single function in response to metabolic stress, oxidative stress, and inflammatory cytokines. Research related to the subtypes of myofibroblasts and their interactions with endothelial cells, cardiomyocytes, immune cells, and resident fibroblasts are emerging with the advance of multi‐dimensional analysis using single‐cell transcriptomics, proteomics and mass cytometry. Future reductionist approaches may be directed toward understanding of the roles of myofibroblast subpopulations in homeostasis, inflammation, fibrosis, tissue healing, transdifferentiation of cell types, and tissue regeneration.

A holistic approach of validating results in autopsy or explanted heart studies and rodent models may be helpful in order to reconcile the differences in pro‐fibrotic responses among species and models of right ventricular pressure overload and considering the variability in number of resident fibroblasts. Some of the difficulties in obtaining human ventricular tissue can be circumvented by obtaining human ventricular cardiac fibroblasts from commercial sources, explanted hearts rejected for transplant, or from autopsies. As stiffness is critical for the transformation of cardiac fibroblasts into myofibroblasts, results from studies using tissue culture dishes require validation in 3D models, artificial matrixes and directional and positional cues in reconstructed tissue injury models. The recent surge in the discovery of immune mediators in the pro‐fibrotic responses in right ventricular pressure overload warrants further investigation in the roles of immune cells and cytokines in cardiac fibrosis and right ventricular dysfunction (Dewachter & Dewachter, 2018; Sydykov et al., 2018).

## 5 ROLE OF IMMUNE CELLS AND CYTOKINES

Inflammatory cells such as mast cells, macrophages, dendritic cells, and B cells and T cells are seen in the perivascular fibrotic regions and have been implicated with worse outcomes in PAH (Heath & Yacoub, 1991; Perros et al., 2012; Perros, Dorfmüller, Souza, Durand‐Gasselin, Mussot, et al., 2007; Price et al., 2012; Tuder, Marecki, Richter, Fijalkowska, & Flores, 2007). Helper T cells stimulate B cells to produce antibodies and activate macrophages; these macrophages are divided into classically activated (M1) or alternatively activated (M2) categories that promote Th1 and Th2 responses respectively (Mills, 2012; Mills, Kincaid, Alt, Heilman, & Hill, 2000). CD4^+^ helper T cells secrete mediators that can interfere with fibroblast and macrophage function and result in progression of fibrosis in lungs (Chakraborty, Chatterjee, & Bhattacharyya, 2018). In the heart, CD4^+^ T cell activity can influence cardiac remodeling and scarring (Ramos, Hofmann, & Frantz, 2016). Depletion of CD4^+^ T cells, the T helper cells, slows the progression of pulmonary arterial muscularization in the lungs (Daley et al., 2008). T‐cell targeted antibodies reverse cardiac fibrosis in systemic sclerosis patients and a mouse model of inflammation associated fibrosis (Crunkhorn, 2016).

**Figure 1:**
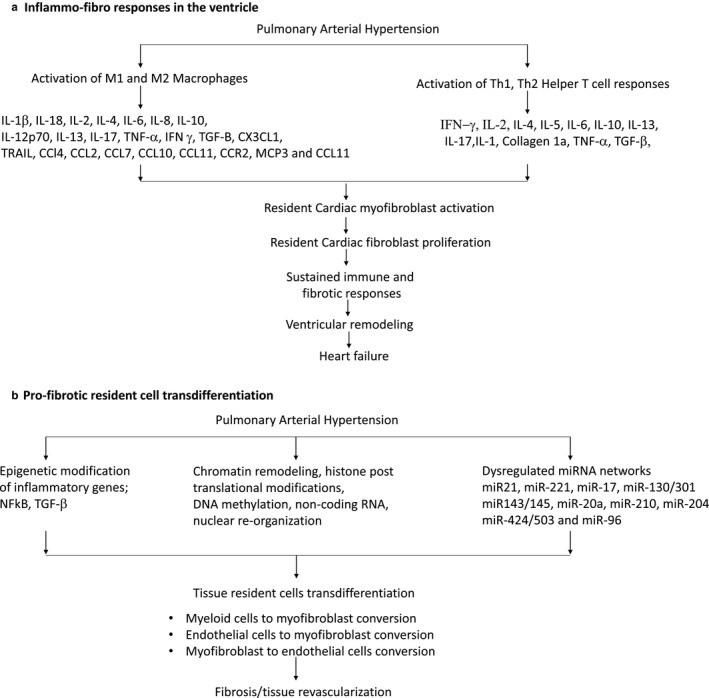
Complexity and interdependence of pathogenic processes in right ventricular fibrosis and pulmonary arterial hypertension

The inflammatory process includes the formation of an inflammasome complex that detects pathogens and activates macrophages to secrete pro‐inflammatory cytokines such as interleukin‐1β (IL‐1β) and IL‐18 as a part of an innate immune response (Figure 1) (Ceneri et al., 2017; Latz, Xiao, & Stutz, 2013). PAH patients have significant increases in circulating IL‐1β and IL‐18 levels when compared to normal healthy patients associated with adverse outcomes (Elaine et al., 2010; Humbert et al., 1995; Ross, Strieter, Fishbein, Ardehali, & Belperio, 2012). IL‐18 increases smooth muscle cell proliferation, contributing to hypertrophy and fibrosis in WHO classified Type I PAH patients (Ross et al., 2012). Additionally, interleukin family cytokines such as IL‐2, IL‐4, IL‐6, IL‐8, IL‐10, IL‐12p70, and tumor necrosis factor‐α (TNFα) are signatures of right ventricular failure in PAH patients (Elaine et al., 2010; Humbert et al., 1995). Transgenic mice overexpressing IL‐6 develop vascular remodeling and PAH through proliferative and anti‐apoptotic mechanisms, when exposed to hypoxic conditions (Steiner et al., 2009). In humans, higher levels of these aforementioned inflammatory cytokines are independently associated with mortality in PAH patients, indicating the significance of macrophage derived cytokines in PAH (Cracowski et al., 2014). This corresponds with a previous study that suggested that increased cytokine levels in blood were correlated with decreased chances of survival at 1‐year, 3‐year, and 5‐year time points in PAH patients (Elaine et al., 2010). These results suggest that cytokine levels are associated with fibrosis and mortality in humans.

Transcriptomic profiling of right ventricle in monocrotaline‐treated male Sprague Dawley rats and comparisons with right ventricle of pulmonary artery banded mice and humans with bone morphogenetic protein receptor type 2 (BMPR2) mutations shows the signatures of mitochondrial dysfunction, fibrosis, angiogenic rarefaction, and inflammation in right heart failure (Potus, Hindmarch, Dunham‐Snary, Stafford, & Archer, 2018). Annexin A1(ANXA1), identified as a key dysregulated gene in decompensated rat hearts, is expressed primarily by leukocytes and inhibits ET‐1 associated inflammatory cytokine secretion (Potus et al., 2018). Proinflammatory gene expression, fibrotic gene expression, and collagen I deposition in the left ventricle increases in ANXA1 knockout mice after myocardial infarction (Qin et al., 2017). These animal studies also indicate the importance of cytokines in sustaining fibrosis leading to ventricular failure.

Elevated levels of chemokines such as Regulated upon Activation, Normal T Cell Expressed and Presumably Secreted (Dorfmüller et al., 2002), fractalkine (CX3CL1) (Perros, Dorfmüller, Souza, Durand‐Gasselin, Godot, et al., 2007), CC chemokine ligand 2 (CCL2) (Sanchez et al., 2007), and CXCL10 (Ross et al., 2012) are also found in patients with PAH. Proteomic profiling of blood biomarkers to identify the immune cell subtype on 281 PAH patient cohorts identified several pro‐inflammatory cytokines, chemokines and growth factors central to immune system upregulation, such as CCL4, CCL5, TNF‐related apoptosis‐inducing ligand, CCL7, Macrophage migration inhibitory factor, and CCL11 (Sweatt et al., 2019). Moreover, researchers have identified clinical risk associated immune phenotypes that are independent of the WHO group 1 subtypes (Sweatt et al., 2019). Altogether, it is unclear if these pro‐inflammatory cytokines and chemokines also participate in right ventricular fibrosis.

## 6 ROLE OF MACROPHAGE SUBTYPES

Cardiac injury, such as myocardial infarction, activates the innate immune response through the elicitation of an inflammatory phase and a reparative phase. Mutually exclusive immune cell populations are the main players of the innate immune response. Pro‐inflammatory lymphocyte antigen 6 rich in cysteine (Ly6C^hi^) monocytes from splenic reservoirs or bone marrow give rise to activated M1 macrophages, which promote inflammation and phagocytosis intended to remove dead and dysfunctional cardiomyocytes (Barnette et al., 2018). The macrophages are then replenished during inflammatory conditions through recruitment of other macrophages in the chemokine receptor type 2 (CCR2) and monocyte chemotactic protein‐3 (MCP3)‐dependent manner (Gerasimovskaya et al., 2012; Stow, Ching Low, Offenhäuser, & Sangermani, 2009). The reparative Ly6^low^ monocytes are associated with alternatively activated M2 macrophages which resolve the inflammation and propagate repair through collagen deposition to maintain the structural integrity of the left ventricle (Barnette et al., 2018; Dewald et al., 2004; Jugdutt, Joljart, & Khan, 1996; Nahrendorf, Pittet, & Swirski, 2010).

While the immune system participates in the resolution of injury, it often “overreacts” to the initial “trigger” by a process of bystander damage to neighboring cells (Epelman, Liu, & Mann, 2015). Recent single cell sequencing studies point to the significant role of tissue resident macrophages in inflammation and tissue repair. Resident macrophages in the heart are distinguished by the cell surface expression of CCR2 (Bajpai et al., 2018). The CCR2^‐^ macrophages enter the heart during embryonic development and in the absence of monocyte recruitment have a role in tissue homeostasis (Lavine et al., 2014; Leid et al., 2016). In contrast, the CCR2^+^ macrophages are recruited after a few weeks of life and are maintained through monocyte recruitment and proliferation (Bajpai et al., 2018). CCR2^+^ macrophages are associated with initiation of inflammation and coronary angiogenesis, tissue repair, and cardiac regeneration (Bajpai et al., 2018). A shift in the balance from CCR2^‐^ to the CCR2^+^ population is seen in patients with left ventricular heart failure (Bajpai et al., 2018). Antibody depletion of CCR2^+^ macrophages alleviates left ventricular remodeling and cardiac fibrosis (Patel et al., 2018). CCL2 global deletion mice have attenuated postinfarct left ventricular remodeling and reduced accumulation of myofibroblasts (Dewald et al., 2005). Macrophage also play a role in adverse remodeling and right ventricular hypertrophy due to pressure overload (Forman, Brower, & Janicki, 2006). Macrophage derived cytokines such as IL‐1β are associated with right ventricle failure and PAH in animal models in dogs, rats and pigs (Belhaj et al., 2013; Dewachter et al., 2010; Rice et al., 2016; Rondelet et al., 2012). Strong evidence suggests the association of plasma macrophage‐mediated inflammatory cytokines such as TNFα, IL‐1β, and IL‐6 with cardiac remodeling such as hypertrophy, fibrosis and apoptosis in heart failure (Gullestad et al., 2012). Fibrosis is a feature of several chronic inflammatory illnesses involving both innate and adaptive immune response (Wynn & Ramalingam, 2012). Inflammation and fibrosis are inter‐related and have many overlapping aspects. Macrophages, T helper cells, and myofibroblasts play important roles in both inflammation and in fibrosis (Lupher & Gallatin, 2006; Wick et al., 2010).

Tissue repair usually begins with inflammation, which involves the recruitment of cytokines and the proliferation and conversion of fibroblasts into myofibroblasts (Lupher & Gallatin, 2006). Myofibroblasts and CD4^+^ T cells secrete collagen and TGF‐β functioning as anti‐fibrotic and anti‐inflammatory cytokine. Reactive fibrosis results from a switch from the initial Th1 inflammatory cell response to Th2 cells with prolonged exposure to an inflammatory stimulus response (Usher et al., 2019). The Th1 response involves the production of IFN‐gamma and IL‐2 production by the macrophages. The Th1 response is generally effective against intracellular parasites. The Th2 cell responses involve production of IL‐4, IL‐5, IL‐6, IL‐10, and IL13 cytokines that participate in tissue healing through collagen deposition and fibrosis. IL‐17, IL‐6, TNFα, and IL‐1 feedback loops are important drivers of chronic inflammatory disease (Usher et al., 2019). TGF‐β, pro‐inflammatory, and pro‐fibrotic molecules are the primary driver of fibrosis in other tissues (Usher et al., 2019). Elevated TGF‐β expression correlates with abnormal connective tissue deposition seen in fibrotic diseases (Usher et al., 2019; Wynn & Ramalingam, 2012).

The macrophage cells produce cytokines such as IL‐1β to initiate inflammation. IL1β belongs to the family of inflammatory cytokines and their receptors share similar innate functions, like pattern recognition, with conserved Toll‐like receptor families (Gerasimovskaya et al., 2012). PAH patients have elevated serum IL‐1β levels (Voelkel, Tuder, Bridges, & Arend, 1994). IL‐1β also accumulates in the right ventricle of patients with PAH (Cracowski et al., 2014; Duncan et al., 2012; Elaine et al., 2010; Humbert et al., 1995; McMahan, Schoenhoff, Van Eyk, Wigley, & Hummers, 2015). Anakinra, an anti‐IL‐1 drug commonly used for rheumatoid arthritis is currently being tested as a repurposed drug for patients with PAH (Trankle et al., 2019).

## 7 EPIGENETIC MODIFICATIONS OF INFLAMMATORY MOLECULES LEADING TO FIBROSIS

Transdifferentiation or phenotype switching involves nuclear reprogramming and epigenetic modifications. Recent epigenetic hypotheses suggest that myofibroblasts inherit an altered phenotype that promotes excessive fibrotic tissue accumulation (Robinson, Watson, & Baugh, 2012). Inherited or de novo epigenetic modifications alter gene expression pattern and activity which, in the aggregate define cellular identities and functions. Epigenetic modifications with potential to treat cardiac fibrosis (Felisbino & McKinsey, 2018) include chromatin remodeling, histone post translational modifications, DNA methylation, noncoding RNA, nuclear re‐organization, and miRNA regulation. Possible epigenetic mechanisms contributing to PAH include dysregulated miRNA networks, DNA methylation, increase in Reactive Oxygen Species generation and aberrant expression of histone deacetylases (Chelladurai, Seeger, & Pullamsetti, 2016; Kim et al., 2015; Zhou, Chen, & Raj, 2015). miRNAs regulate gene expression, and dysregulation is important to the development of PAH (Spiekerkoetter, Kawut, & de Jesus Perez, 2019). Possible miRNA related therapies include inhibitors of miR‐17, miR‐130/301, miR‐143/145, miR‐20a, and miR210 and mimics of miR‐204, miR424/503, and miR‐96 (Chun, Bonnet, & Chan, 2016). A recent paper indicates that RV fibroblasts undergo chamber‐specific mitochondrial epigenetic reprogramming to promote RV fibrosis, RV hypertrophy, and RV failure (Tian et al., 2020).

Chronic inflammation causes epigenetic modifications and activation of myofibroblasts (Usher et al., 2019). A study of a mouse model of kidney fibrogenesis demonstrates that epigenetic modifications may be one of the several reasons behind fibroblast activation (Bechtel et al., 2010). Histone acetylation, an epigenetic modification, activates inflammatory genes (Bayarsaihan, 2011). Airway biopsies and alveolar macrophages from chronic obstructive pulmonary disease indicate that histone acetylation of inflammatory genes is mediated by NF‐κβ (Bayarsaihan, 2011). TGF‐β, another pro‐inflammatory molecule, also increases methylation of anti‐fibrotic genes and decreases methylation of fibrotic genes (Usher et al., 2019). DNA methylation increases are also associated with fibrosis of the heart (Felisbino & McKinsey, 2018), lungs (Evans et al., 2016), and other organs (Meng, Nikolic‐Paterson, & Lan, 2016). Activation of myofibroblasts can trigger histone acetylation, which strengthens the pro‐fibrotic effects of epigenetic modifications (Usher et al., 2019). Epigenetics control pro‐inflammatory TNFα, interleukins, autocrine, and paracrine activation of the transcription factor NF‐κβ thereby contributing to the inflammatory response (Shanmugam & Sethi, 2013). Therefore, epigenetic changes should be taken into consideration when designing novel therapeutics for right ventricular fibrosis and PAH.

## 8 CONCLUSION

Pulmonary arterial hypertension is a complex disease with multiple, inter‐dependent mechanisms known to be involved in the initiation and propagation of ventricular dysfunction (Figure 1). Although currently available therapies can alleviate PAH symptoms, there is presently no cure for a failing right ventricle. Current research is expanding our understanding of innate immune responses and development of fibrosis and right ventricular failure. Knowledge of cardiac fibroblast and immune cell interactions in orchestrating inflammation and resolution of inflammation will be key to preventing exacerbated immune and fibrotic responses. Identification and characterization of immune cell subtypes and cardiac fibroblast cell subtypes associated with cardiac fibrosis and right ventricular remodeling present new therapeutic opportunities. Resolution of the roles of tissue resident and transdifferentiated cardiac fibroblasts will provide a clearer picture of the specific cardiac fibroblast subtypes involved in progression of right ventricular failure in PAH. The heterogeneity and myriad roles of cardiac fibroblasts are coming to light through newer technologies such as single cell sequencing of tissue resident cells and their roles in inflammation and fibrosis determined. Blood based biomarkers and high throughput screening technologies can be used for risk stratification, disease monitoring, and prognostication. Genetic tools are needed to understand the time dependent diverse phenotypes of cardiac fibroblasts at genomic, proteomic, metabolomic levels in settings of inflammation and fibrosis in order to formulate treatments to prevent immune‐induced right ventricular fibrosis and PAH.

## CONFLICTS OF INTEREST

The authors declare no conflicts of interest.

## AUTHOR CONTRIBUTIONS

JHS and SR conceived the ideas in the review paper. JHS, AZ and HB participated in writing the manuscript. FSP designed the schematic figure. SR contributed to the ideas, significantly edited the paper and substantially revised it. RJG contributed to the writing and editing of the paper. All the authors have read and approved the manuscript.
